# Retraction Note: Quality of life and its correlated factors among patients with substance use disorders: a systematic review and meta-analysis

**DOI:** 10.1186/s13690-026-01971-7

**Published:** 2026-05-29

**Authors:** Bahram Armoon, Marie-Josée Fleury, Amir-Hossien Bayat, Azadeh Bayani, Rasool Mohammadi, Mark D. Griffiths

**Affiliations:** 1 Douglas Hospital Research Centre, Montreal, QC Canada; 2 Department of Psychiatry, McGill University, Montreal, QC Canada; 3 Social Determinants of Health Research Center, Saveh University of Medical Sciences, Saveh, Iran; 4 Student Research Committee, School of Allied Medical Sciences, Shahid Beheshti University of Medical Sciences, Tehran, Iran; 5 Department of Biostatistics and Epidemiology, School of Public Health and Nutrition, Lorestan University of Medical Sciences, Khorramabad, Iran; 6 International Gaming Research Unit, Psychology Department, Nottingham Trent University, Nottingham, UK

**Retraction Note: Arch Public Health 80, 179 (2022)**

**https://doi.org/10.1186/s13690-022-00940-0**

The Editors-in-Chief have retracted this article. After publication the Editors-in-Chief were made aware that this article contains material that substantially overlaps with [1]. Independent post-publication peer review has confirmed, among others, the following issues:

- the result of the publication bias test is identical with the corresponding test in [1], despite there being no overlap in the final set of articles included in this meta-analysis and in [1];
- in the PRISMA flowchart, the number of articles identified (including from each database), the number of duplicates removed and the number of papers screened are identical with the number of articles in the corresponding stages in the PRISMA flowchart of [1];
- overlap of text with [1], particularly in the Abstract, Background and Methods sections.

The Editors-in-Chief therefore no longer have confidence in the validity of the data and the conclusions drawn.

Bahram Armoon, Marie-Josée Fleury, Azadeh Bayani, and Mark D. Griffiths disagree with this retraction. Rasool Mohammadi has not responded to correspondence regarding this retraction. The Publisher has not been able to obtain a current email address for Amir-Hossien Bayat.
